# Maternal Mortality-reduction Programme in Andhra Pradesh

**DOI:** 10.3329/jhpn.v27i2.3365

**Published:** 2009-04

**Authors:** M. Prakasamma

**Affiliations:** Academy for Nursing Studies, Flat 215, Amruthaville Apartments, Rajbhavan Road, Hyderabad 500 082, Andhra Pradesh, India

**Keywords:** Health facilities, Maternal health, Maternal mortality, Safe motherhood, India

## Abstract

Andhra Pradesh, a large state in southern India, has a high maternal mortality ratio of 195 per 100,000 livebirths despite the improvements in social, demographic and health indicators over the last two decades. This contrary situation has been analyzed using findings of different studies on maternal mortality, and four factors have been presented for consistently-high maternal mortality in the state. First, the disproportionately-high focus on family planning towards population stabilization reduced the emphasis on maternal health in the peripheral hospitals, resulting in low use of these facilities for childbirths. Second, the growth of services in Primary Health Centres was not given adequate emphasis, resulting in the weakening of the peripheral health system. Third, there was little emphasis on developing a cadre of midwives who would have primarily focused on maternal health. Lastly, the low status of women in the state has hampered timely referral and access to services.

## INTRODUCTION

### Profile of Andhra Pradesh

Andhra Pradesh, one of the 28 states of India, is located in the southeastern part of the country with Karnataka in the West, Tamil Nadu in the South, Maharashtra, Chattisgarh, and Orissa in the North, and the Bay of Bengal in the East. Its total land area is 275,045 sq km with a long coastline of about 1,000 km. The state is ranked fifth in the country in terms of its area and population size.

According to the Census 2001, the total population of Andhra Pradesh was 75,727,541. The density of population is 277 per sq km compared to 312 in the country. The state has 23 districts, including the capital district Hyderabad. It is divided into three sociocultural and geographic regions—Coastal Andhra, Telangana, and Rayalaseema—with 10 districts, including Hyderabad in the Telangana region, nine districts in the Coastal region, and four districts in the Rayalaseema region. Andhra Pradesh is predominantly rural and agricultural, with 73% of the total population living in 28,123 villages and hamlets, according to the Census 2001 (1).

Andhra Pradesh fares better than other states on key demographic indicators (Table 1). Its population is growing at a slower decadal growth rate at 14.59% compared to 21.54% in the rest of India. The contraceptive prevalence rate (CPR) is 68% according to the National Family Health Survey (NFHS) 3 compared to the national average of 56.3% (2). The total fertility rate (TFR) in Andhra Pradesh is 1.8 compared to 2.9 in the country. Overall, the demographic profile indicates that the state has moved into the next phase of demographic transition.

**Table 1.** Demographic, social and health profile of Andhra Pradesh compared to national average

Characteristics	Andhra Pradesh	India
Total population (Census 2001) (million)	76.21	1,028.61
Percentage of urban population	27.3	27.8
Crude birth rate (SRS 2006)	19	24.1
Crude death rate (SRS 2006)	7	7.5
Decadal growth (Census 2001) (%)	14.59	21.54
Total fertility rate (SRS 2004)	1.8	2.9
Sex ratio—no. of females per 1,000 males (Census 2001)	978	933
Contraceptive prevalence rate (NFHS 3)	67.7	56.3
Population below the poverty-line (%)	15.77	26.10
Human development index (NHDR 2001)	0.377	0.38
Human poverty index (NHDR 2001)	39.78	39.36
SC population as a % of total population (Census 2001)	16.20	16.2
ST population as a % of total population (Census 2001)	6.59	8.2
Female literacy rate (Census 2001) (%)	50.4	53.7
Mean age (years) at effective marriage for girls (SRS 1999)	18.1	19.6
Infant mortality rate (SRS 2006)	59	58
Maternal mortality ratio (SRS 2001-2003)	195	301

NFHS=National Family Health Survey; NHDR=National Human Development Report; SRS=Sample Registration System; SC=Scheduled castes; ST=Scheduled tribes

Over the last six decades, Andhra Pradesh has made great leaps in areas, such as information technology and population stabilization. In other areas, trends do not show much improvement, especially in terms of health of infants and status of women. Sex ratio in the state is slightly better than the national average with 978 females to 1,000 males compared to 933 in the rest of India. Infant mortality is nearly the same as the rest of India. In the area of maternal health, Sample Registration System (SRS) data show that the state has a lower maternal mortality ratio (195 per 100,000 childbirths) compared to the national estimate of 301 (3). The percentage of population below the poverty-line is lower in the state (15.77%) compared to the country as a whole (26.1%). However, the human development index and the human poverty index of the state are not encouraging and are similar to the national average according to the National Human Development Report (NHOR) 2001 (4). In some critical areas of social development of women, the state falls below the national average. Female literacy and age at marriage are two examples where Andhra Pradesh lags behind the country as a whole. Only half of the females aged seven years and above are literate in the state. The mean age at effective marriage for females in Andhra Pradesh according to the 1999 SRS is 18.1 years—about one and a half years lower than the national figure of 19.6 years.

**Fig. 1.**
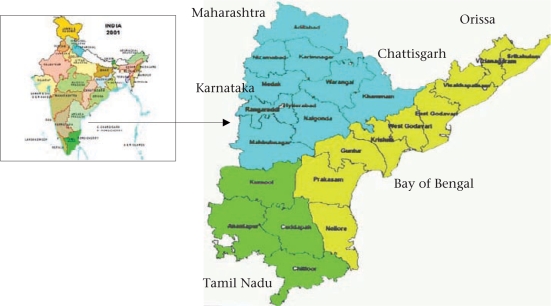
Map of Andhra Pradesh

This case study was carried out with the following three objectives: (a) analyze the slow reduction of maternal mortality in Andhra Pradesh in contrast to achievement of demographic goals and in comparison with the neighbouring states—Tamil Nadu and Kerala; (b) contextualize the high maternal mortality ratio (MMR) within the sociodemographic and programmatic scenario of the state and assess the factors contributing to unsatisfactory achievements in the area of maternal health compared to huge successes in the area of population stabilization in terms of strategies chosen and opportunities missed; and (c) design and implement sustained advocacy for cost-effective and comprehensive strategies for achieving safe motherhood, including pilot interventions to showcase the best practices.

## MATERIALS AND METHODS

The background work for this case study included analysis of qualitative and quantitative data, opinions of stakeholders, and advocacy meetings over a three-year period from 2005 to 2007. Both primary and secondary sources were used for analysis.

Two primary sources used were:

- An indepth study of maternal health services was conducted in one district—Medak—to gain specific insights into the functioning of the public-health system for maternal health services at the peripheral level.
- Tracking and auditing of 148 maternal deaths reported in 2006 in Andhra Pradesh was done to understand the contributory factors.

### Indepth study in Medak district

Medak district was selected because it fell below the state average on most indicators, such as literacy, female age at marriage, crude birth rate (CBR) (23.3), TFR (2.9), and female literacy (38.7%) (Table 2).

**Table 2.** Demographic profile of Medak district

Demographic profile	Medak	Andhra Pradesh
Urban population (%)	14.4	27.3
CBR	23.3	19.0
TFR	2.9	1.8
Female Literacy Rate	38.7	50.4

CBR=Crude birth rate; TFR=Total fertility rate

Medak is a large district (9,700 sq km), located in the Telangana area, with a population of 2,670,097, according to the Census 2001. The district is predominantly rural and agricultural with an urban population of only 14.4%. There are 45 mandals (smaller than blocks), 11 towns, and 1,225 villages. The medical and health infrastructure of the district consists of one district hospital, three area hospitals, and 10 Community Health Centres (CHCs). Primary healthcare is provided through 64 Primary Health Centres (PHCs) and 489 Subcentres. There is a wide network of private health facilities with two medical colleges attached with teaching hospitals and 120 nursing homes, or smaller hospitals. The district designated 26 of its 64 PHCs as round-the-clock centres for maternal and child health emergencies. Moreover, the Academy for Nursing Studies initiated a project in 2007 to pilot-test the operationalization of the 24-hour PHCs in the district in collaboration with the Government of Andhra Pradesh.

### Methods used for data collection

Data were collected on facilities in hospitals and health centres, perceptions of women, and perceptions, knowledge, and skills of care providers. Trained midwifery and public-health staff made observations and conducted focus-group discussions and interviews. For facility assessment, a checklist was prepared after discussion and inputs from midwifery and public-health experts for facility assessment of the 26 PHCs providing 24-hour service and 10 CHCs in Medak. Skill-management exercise was conducted for staff nurses, Auxiliary Nurse Midwives (ANMs), and medical officers in these facilities on key areas of the maternal and child health checklist. Interviews were also conducted with 15 medical officers, 41 staff nurses, and 28 ANMs in these facilities (26 PHCs and 10 CHCs).

Focus-group discussions were conducted with health professionals. Separate guidelines were followed for different medical professionals, viz. medical officers, ANMs, and staff nurses. Focus-group discussions were also held with women's groups in four villages.

### State-wide tracking of maternal deaths

The Sample Registration System estimated the MMR to be 195 per 100,000 livebirths (3). This means that about 3,000 maternal deaths take place for the nearly 16,00,000 livebirths in the state each year. Repeated requests to the district health officials and others (healthcare providers, district administrators, women health volunteers, and NGOs) revealed only a small portion of the expected deaths in each district. There was no uniformity in numbers. Only about 10% of the estimated maternal deaths in Andhra Pradesh were identified for 2006 up to February 2007, indicating the low priority for reporting and registering maternal deaths. Of 336 deaths identified, the research team conducted a review for 148 maternal deaths that could be traced back to their houses. The full details of the remaining maternal deaths were not available for analysis. State-level primary data on maternal deaths and the factors contributing to deaths were obtained from social audits conducted for these 148 deaths.

A detailed analysis of secondary data relating to maternal deaths, availability of health services, use of facilities, and government interventions in the state during the last two decades was conducted. Some reports used were: three rounds of the NFHS, SRS data, census data, and departmental reports of the Government of India and the Government of Andhra Pradesh. Findings of research and project reports conducted in the state were also used. Programme-related data for the last 20 years were collected, reviewed, and analyzed and were included in the paper wherever applicable.

## RESULTS

Results of the study are presented under the following three sections:

- Infrastructure, facilities, and personnel for maternal health service-delivery in Andhra Pradesh and the awareness and use of facilities by the community, specifically women.
- Maternal mortality in Andhra Pradesh, including efforts at estimation, the medical causes, and social factors contributing to maternal deaths.
- Government interventions for reducing maternal mortality and morbidity, including different programmatic efforts over the last two decades.

**Infrastructure, facilities, and personnel for ma ternal health services and awareness and use of facilities by the community, specifically women**

Started in the erstwhile Hyderabad state under the Nizam rule before the independence of India, the tradition of maternal and child health service-delivery continued after the merging of Hyderabad into the Indian Union. The state followed the same pattern of community development as in the rest of the country. A steady growth of public-health institutions took place—Subcentres, PHCs, and hospitals increased in number. A number of training centres were established to produce doctors, nurses, health visitors, and ANMs to work in these health centres. However, maternal health services gradually took a back seat in the priorities of the health system. Maternal and child health became integrated into family planning, following national guidelines that placed primary emphasis on measures to control population growth.

### 

#### Structure of health services in Andhra Pradesh

Andhra Pradesh has a wide network of health facilities organized in a three-tier manner—primary, secondary, and tertiary—each managed by a different administrative structure within the health department.

• The Directorate of Health is responsible for preventive, promotive and basic curative healthcare services throughout the state. Primary health services are provided under this system through a network of 1,570 PHCs and 12,522 Subcentres (Table 3). The Rural Health Statistics Bulletin, 2007 reported that each PHC covers a population of 35,287 and an area of 172.16 sq km, on an average, supports about 8-10 Subcentres, and provides institution-based primary medical care, maternal and child care, immunization and family-planning services (5). Each Subcentre covers a population of 4,424 and an area of 21.59 sq km and provides outreach services. Over the last two decades, the number of Subcentres doubled from 6,125 to 12,522, and the number of PHCs rose three times from 555 to 1,570.
  **Table 3.** Growth of health facilities in Andhra Pradesh, 1981-2005

Period	No. of Subcentres	No. of PHCs
1981-1985	6,125	555
1985-1990	7,894	1,283
1992-1997	10,568	1,335
1997-2002	10,568	1,386
Functioning as on September 2005	12,522	1,570

PHCs=Primary Health Centres
• Although the number of health facilities has increased in the state, there is a shortage of staff in these facilities, especially nurse-midwives according to the above bulletin. Moreover, the requirement is itself below the optimum—2,739 nurse-midwives for 1,924 PHCs—i.e. less than two nurse-midwives per PHC (Table 4). This indicates that adequate staff positions were not created for their round-the-clock functioning. This has been further highlighted in the case study of Medak district later in the paper.
  **Table 4.** Primary health infrastructure and key health personnel in Andhra Pradesh, 2006

Particulars	Required	In position	Shortfall	% of shortfall
Subcentres	11,699	12,522	-	-
Primary Health Centres	1,924	1,570	354	18.40
Multipurpose workers (female)/ANMs	14,092	13,740	352	2.50
Health Assistants (female)/LHVs at PHCs	1,570	1,564	6	0.38
Doctor at PHCs	1,570	2,202	-	-
Nurse/midwives (staff nurses)	2,739	2,053	686	25.05

ANMs=Auxiliary Nurse Midwives; LHVs=Lady Health Visitors; PHCs=Primary Health Centres
• The Andhra Pradesh Vaidya Vidhan Parishad (APVVP) was established in 1986 under an act of legislation with the objective of strengthening the secondary-level health system. The APVVP deals exclusively with hospitals of bed-strengths ranging from 30 to 350 which are referred to as secondary hospitals or first-level referral hospitals. At present, there are 288 hospitals under the control of APVVP, of which 23 are district hospitals, 58 are area hospitals, and 167 are CHCs. The average bed-strength is 200-350 in the district hospitals, 100 in the area hospitals, and 30-50 in the CHCs. Besides, there are 10 specialty hospitals and 25 dispensaries. The CHCs are the First Referral Units (FRUs) at the periphery. These are expected to provide round-the-clock comprehensive obstetric and neonatal services, including emergency management. Table 5 shows that there is an acute shortage of all categories of service providers at this level according to reports obtained from the Government of Andhra Pradesh.

**Table 5.** Health facilities and care providers in Community Health Centres, 2007

Particulars	Required	In position	Shortfall	% of shortfall
Community Health Centres	481	167	314	65.28
Obstetricians and gynaecologists	167	73	94	56.29
Physicians	167	42	125	74.85
Paediatricians	167	54	113	67.66

Source of data: Reports obtained from the Andhra Pradesh Vaidya Vidhan Parishad, Government of Andhra Pradesh
• The Directorate of Medical Education is the administrative authority for functioning of medical colleges, attached teaching hospitals, nursing schools, and dental colleges in the state. There are 35 teaching hospitals with a total bed-strength of 13,606. Half of these beds are in six large general hospitals. The Osmania General Hospital in Hyderabad and the Gandhi Hospital in Secunderabad together serve more than 5,000 outpatients and about 300 emergency patients daily. In 1986, a university of health sciences was established at Vijayawada—(later named NTR University of Health Sciences)—and all university-level institutions of allopathic and Indian systems of medicine, dentistry, and nursing were brought under its academic control.
• The Commissionerate of Family Welfare implements all family-planning activities, including the national Reproductive and Child Health (RCH) programmes and the National Rural Health Mission through the primary health facilities. The department also has the responsibility for in-service training of field-level health and family welfare workers, such as ANMs and male health workers.

#### Healthcare awareness and use of maternal services in Andhra Pradesh

**Fig. 2.**
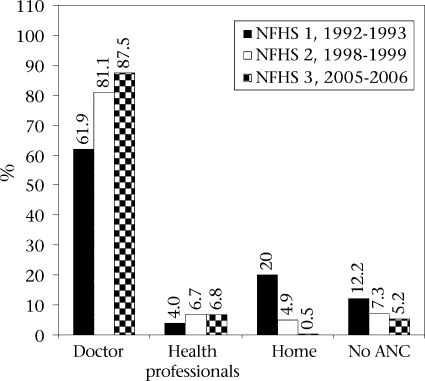
Perentage of women reeing antenatal are from different proiders

**Fig. 3.**
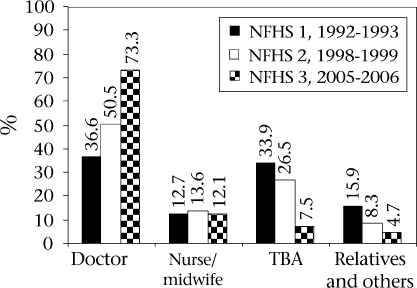
Assistance during delivery

Health awareness and use of maternal healthcare services have shown a shift in the last two decades. There is a clear move from home to institutions and from traditional to professional providers for both antenatal and delivery care as indicated in Figure 2 and 3. During 1992-2005 (NFHS 1, NFHS 2, and NFHS 3), antenatal care from health professionals, especially doctors, increased, and the percentage of women who availed of antenatal care at home from traditional birth attendants (TBAs) reduced sharply (6,7). The percentage of women who did not avail of any antenatal care fell from 12.2% to 5.2 % during this period.

ANC=Antenatal care; NFHS=National Family Health Survey

NFHS=National Family Health Survey; TBA=Traditional birth attendant

Deliveries attended by doctors rose from 36.6% during NFHS 1 to 73.3% during NFHS 3 but deliveries attended by nurses-midwives did not undergo a change (Fig. 3). During the same period, the share of deliveries attended by *dais* fell from 33.9% to 7.5%, and those attended by relatives and others fell from 15.9% to 4.7%.

#### In-depth study of maternal health facilities and services in Medak district

The work in Medak carried out by the Academy for Nursing Studies up to December 2007 illustrates that none of the 26 PHCs that were to be open on a 24-hour x 7-day basis was adequately staffed or equipped for round-the-clock maternal and neonatal services (8) (Table 6). The Indian public health standards of 2006 for PHCs require two doctors and three staff nurses for round-the-clock services (9). These were managed with a few basic instruments as delivery-sets were not available. The labour-rooms were disorganized and dusty; some had cobwebs. Even basic equipment, such as blood pressure apparatus, had to be shared with the ward. There was very little consideration for privacy with broken windows and no curtains. Staff positions were particularly inadequate. The infrastructure was inadequately used. The average number of deliveries per month was 11 (no deliveries were being conducted in four of the 26 PHCs).

**Table 6.** Shortfall of personnel in Medak district, 2007

PHC personnel	Required according to IPHS	Available	% of shortfall
2 medical officers per PHC	52	23	55.76
3 staff nurses or nurse-midwives per PHC	78	32	58.97

IPHS=Indian public health standards; PHC=Primary Health Centres

Perceptions and opinions of care providers and women revealed several problems. Staff nurses in the 24-hour PHCs had difficulties in managing childbirth services since they had to carry out many other activities and could not provide round-the-clock coverage. They neither received technical guidance and support nor had adequate equipment and supplies. They had to stay alone during night duties with poor security. No vehicle was available to refer emergencies. Hospital staff did not give priority to women even when staff nurses referred them. Doctors in the 24-hour PHCs stated that equipment was not available so they could not provide quality service. They complained that villagers brought women in labour in the last minute, pleaded to conduct delivery in the PHC, and created a problem if anything went wrong. They also reported that it was difficult for them to be available round-the-clock when there were no facilities to stay. Women stated that they did not go to the PHCs for childbirth since there were no service providers or facilities.

### Maternal mortality in Andhra Pradesh

#### Estimating MMR

The problem of high MMR continues in Andhra Pradesh despite the increase in health infrastructure. There is still no clear figure of how many women actually die or face severe complications, or survive but suffer life-long morbidity due to poor-quality care. Due to the difficulty in studying and calculating the MMR accurately, there are huge disparities in the reported MMR figures for India and for Andhra Pradesh. Disagreement prevails among surveys, national registration systems, and individual researchers of the estimated statistics on maternal deaths. However, there is an agreement among health professionals, researchers, NGOs, and programme managers on two issues—that MMR is higher than reported and that there are critical gaps in information due to flaws in monitoring, recording, and reporting morbidity and mortality.

Estimates of MMR from some studies on maternal deaths in Andhra Pradesh, particularly, findings of two similar studies in the state, need mention. The study conducted in 1984 in Anantapur district gave a figure of 830 (10). A similar study conducted 20 years later in rural Medak district reported a figure of 431 (11). Although findings of other studies were reported, the findings cannot be compared due to the differences in study site or methodology. Mahapatra and others studied causes of death patterns in Andhra Pradesh using the sisterhood method and estimated 256 per 100,000 livebirths (12). A survey of maternal deaths in five districts of Andhra Pradesh during 1995-1996 gave a figure of 712 for the survey area with wide variations among the five districts (13).

#### Medical causes of maternal deaths

Bleeding and eclampsia persist as the two main causes of maternal deaths in Andhra Pradesh. The early classic study of maternal deaths in Anantapur district by Bhatia in 1984 showed puerperal sepsis as responsible for the highest percentage (30.5%) of maternal deaths (10). Results of the study in Medak 20 years later in 2003 showed that pregnancy-induced hypertension, including eclampsia, was responsible for the highest percentage (28.86%) of maternal deaths while sepsis was responsible for only 14.75%. Over time, sepsis as a cause gradually declined with increasing use of antibiotics and better hygiene as a result of promotion of ‘five cleans' during childbirth. Mahapatra and others in 2000 listed abortion and sepsis as two major causes of 52,158 deaths of women aged 15-44 years (12). Haemorrhage continues to contribute a large proportion of maternal deaths—about one-fifth of the women die due to haemorrhage.

#### Social factors contributing to maternal deaths

The clustering of maternal deaths in socially-vulnerable groups was a striking finding of a study in Mahaboobnagar and Medak districts in Andhra Pradesh (14). The study compared 100 normal childbirths with 100 survivors of complications and 50 cases of maternal deaths. Nearly three-quarters (70%) of women who died had no formal education compared to 44% among normal cases. Only 20% of women who died had studied up to primary level compared to 51% of survivors. There were nearly twice as many women belonging to the scheduled castes (SCs) and scheduled tribes (STs) (socially-underprivileged groups) among death cases (48%) compared to survivors (25%) or women who delivered normally (27%). Findings of the study on maternal deaths in five districts of Andhra Pradesh also showed that women from scheduled castes and tribes were the greatest affected, and they together constituted half (50.74%) of maternal deaths identified in the study (13).

The ability to obtain treatment for complications was observed as a major difference between survivors and victims of maternal deaths in a study of factors contributing to maternal deaths conducted in three states: Andhra Pradesh, Madhya Pradesh, and Orissa, coordinated by the Foundation for Research in Health System in 2002 (15). As part of this study, 170 maternal deaths were compared with 300 survivors of maternal complications. Based on in-depth interviews and social audits in the homes of victims and survivors, common complications were reported by the surviving women or by relatives of those who had died, such as postnatal fever, prolonged labour, oedema, and postnatal bleeding. Prolonged labour and convulsions were the major causes of death in the case of victims, and abnormal position and prolonged labour were the complications in the case of survivors. It was reported that survivors were successful in seeking and obtaining treatment in time compared to death cases.

Of the 148 deaths followed-up as part of this case study, 66.2% died after reaching the hospital, 20.3% died on way to the hospital, and 13.5% died at home. This indicates the need for early recognition of problems and decision-making and referral at the periphery.

A maternal death has implications for the health and survival of the newborn and the care and support that the child receives later in life. The 1997 study in five districts found that, of 82 liveborn babies of mothers who had died, 39 died within a few days after the death of the mother. Another component of this study was to follow up the families for six months after the death of the woman. Of the 48 husbands of women whose case studies were written, 25 had married within six months after the death of the wife. The surviving children of the men who had remarried were found in the care of grand parents or other relatives.

### Government initiatives to reduce MMR

Andhra Pradesh has been foremost at designing and implementing health and family welfare programmes, such as formulating a state population policy (1997); establishing a university of health sciences (1986); creating a separate department for first-level referral units, etc. The state has recently initiated several innovative schemes for reducing the number of maternal deaths, such as facilitating the availability of 108 emergency vehicles for rural health services (16). This initiative is treated as a model and is now being emulated in other states. The support to women's groups during the 1990s and the ASHA training programme during this decade are described as exemplary programmes of the state. Most notable is the state's achievement in population stabilization. Some initiatives of the state for reduction in MMR are discussed below.

#### Initiatives to deal with maternal emergencies and complications

Ensuring efficient transport to hospital and availability of blood are two factors for addressing maternal emergencies. Under the National Rural Health Mission and RCH 2, the Family Welfare Wing of the Health, Medical and Family Welfare Department of the state is implementing the Rural Emergency Health Transportation Scheme (REHTS) to provide vehicle facility to rural people for emergencies relating to pregnant women, infants/children, and accidents. The intervention was first pilot-tested in four districts through NGOs but did not show much success when many organizations were involved. In the second phase, the entire operation was handed over to one organization—Emergency Management and Research Institute (EMRI)—operated by a foundation set up by a corporate agency. In total, 502 ambulances have been operationalized in the entire state, each vehicle covering a population of 130,000 to 150,000. There is one central call number (108) to avail of an ambulance. This highly successful intervention is taken as a model for other states.

The problem of availability of blood is also being addressed to some extent. A memorandum of understanding was signed with the Red Cross Society of Andhra Pradesh to upgrade and maintain blood-banks and blood-storage centres in the state. There are still no clear data on how well they are maintained. In the meantime, the number of blood-banks in the private sector and those operated by trusts have increased. Both these interventions are beginning to have an impact on service-use.

#### Efforts for enhancing awareness among women and use of health services

The Government of Andhra Pradesh initiated several schemes and programmes for enhancing the use of health services from public and private hospitals during pregnancy and childbirth. The Women Health Volunteers (ASHA) programme, a component of RCH 2 (17) needs to be specially mentioned. During 2005-2006, 55,400 Women Health Volunteers were identified by the respective *gram panchayats*, and 49,000 received training for one month each until February 2007. A three-day refresher training is planned to be conducted every six months. The State Nodal Agency nominated the Academy for Nursing Studies for designing and conducting the training. This scheme is expected to enhance public awareness and increase demand for services. The Woman Health Volunteer is provided a small remuneration according to her performance. The scheme favours services to vulnerable groups, such as women from the SC and ST population.

*Janani Suraksha Yojana* (JSY) provides care to rural poor women who undergo institutional deliveries whether in a public or private hospital. This programme, implemented with support from the Government of India, has proved to be effective in promoting the shift to hospitals for delivery. Unlike in other states, this money is not paid to women who deliver in the home. The ASHA programme and the JSY programme continue the practice of monetary incentive for performance and service acceptance that were successful in achieving the sterilization targets.

The Government of Andhra Pradesh also launched a Free Bus Pass Scheme under which nearly 0.8 million rural pregnant women received free bus passes to enable them to travel to the nearest hospital for antenatal care and delivery. Another recent development is the Health Information Helpline. A toll-free number (104) has been dedicated to answer calls of people at all times round-the- year through a public-private partnership with the Health Management Research Institute of the Satyam Foundation.

## DISCUSSION

### Analysis of factors responsible for maternal mortality in Andhra Pradesh

The major factors for the persistently high MMR are the low awareness among women and families about risks and low access to resources and services. The failure of the public-health system at the grassroots level to provide a skilled birth attendant for every childbirth is also a critical factor in the current high infant mortality rate (IMR) and MMR as shown in the case study of Medak. In addition, the gradual but steady withdrawal of skilled services and service providers from the community to more centralized urban areas has reduced access to qualified care for underprivileged groups. This case study postulates the following key hypotheses for the persistently high MMR and then discusses each briefly:

• Low priority to maternal health due to disproportionately heavy focus on female sterilization resulted in inadequate efforts at reducing maternal mortality, especially in the last decade. This included low emphasis on monitoring, reporting, and analyzing maternal deaths.
• Inadequate emphasis on strengthening peripheral health facilities resulted in negligence towards and decline of primary health facilities, and women gradually moved away from government facilities.
• Low priority to development of midwifery personnel resulted in extreme shortages in skilled maternal care providers in the hospital and community.
• The low status of women—low age at marriage and low literacy levels of girls—did not generate demand for high-quality maternal health services at the periphery and delayed prompt treatment-seeking.

#### Low priority to maternal health compared to population control

**Fig. 4.**
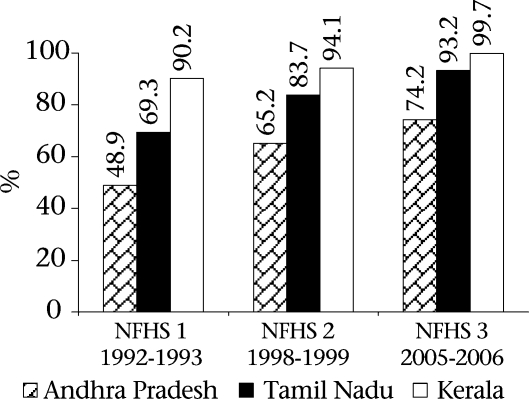
Births assisted by a doctor/nurse/LHV/ANM/other personnel

**Fig. 5.**
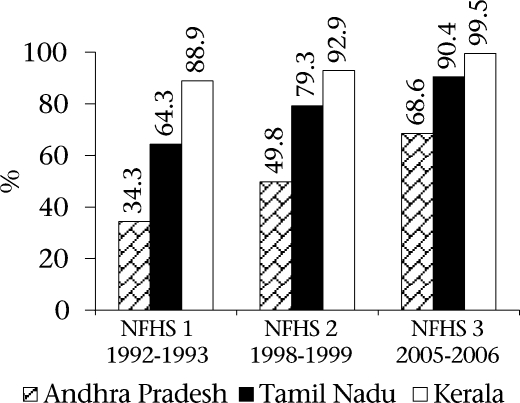
Perentage of institutional births in different states

The findings of the NFHS 3 (2005-2006) indicate that Andhra Pradesh is ahead of the country as a whole in terms of provision of maternal healthcare but is lagging behind Kerala and Tamil Nadu. Figure 4 and 5 depict the situation of maternal health services in the three states. The data are especially striking in the percentage of deliveries conducted in institutions and assisted by health professionals. Only 68.6% of women in Andhra Pradesh delivered in institutions compared to 99.5% in Kerala and 90.4% in Tamil Nadu according to the NHFS 3 data. Although there is a steady improvement in Andhra Pradesh in the percentage of deliveries attended by health personnel, the figure of 74.2% in Andra Pradesh is far behind Tamil Nadu (93.2%) and Kerala (99.7%).

ANM=Auxiliary Nurse Midwife; LHV=Lady Health Visitor NFHS=National Family Health Survey

NFHS=National Family Health Survey

On the other hand, the data in Table 7 show that Andhra Pradesh has moved far ahead of Kerala and Tamil Nadu in female sterilization. It also shows a further decline in the TFR. This clearly suggests that the achievement of demographic goals received higher emphasis compared to maternal health services in Andhra Pradesh.

**Table 7.** Demographic and family-planning indicators of Andhra Pradesh compared to Kerala, Tamil Nadu, and all-India, 1992-2006

Indicator	NFHS	Andhra Pradesh	Tamil Nadu	Kerala	India
Total fertility rate	1	2.59	2.48	2.00	3.39
	2	2.25	2.19	1.96	2.85
	3	1.79	1.80	1.93	2.68
Contraceptive prevalence rate (%) of women using any modern method	1	47.0	45.2	54.4	36.5
	2	58.9	50.3	56.1	42.8
	3	67.0	60.0	57.9	48.5
% of women using female sterilization	1	38.3	37.6	41.8	27.4
	2	52.7	45.2	48.5	34.1
	3	62.9	55.0	48.7	37.3

NFHS=National Family Health Survey

Andhra Pradesh was the first state to formulate the state population policy in 1997 much ahead of the National Population Policy in 2000. The policy starts with a comparison of the demographic situation in Andhra Pradesh with that of Kerala and Tamil Nadu and aims at doing better than the other two states (18). The policy states, “In the short term, the state will aim at reaching the All-India demographic goals set for 2000 AD; and in the long term, it will aim for a Kerala-like situation where the third stage of demographic transition has been reached—low population growth with low fertility and mortality” (p. 5). The policy says that the number of terminal method-users (those who underwent sterilization) in Andhra Pradesh is one of the highest in India with 8 per 1,000 compared to 6 in Tamil Nadu and 4 in Kerala.

“Population control remains the most challenging task before our nation and our state today. This policy document is a statement of the resolve by the Government of Andhra Pradesh to bring about a change in the size, structure and distribution of the population to a level that will result in improving the standard of living and quality of life of the people in general and, in particular, in extending the benefits of such change and development to the most vulnerable and disadvantaged …. Fertility reduction is at the heart of the development of the State,” stated N. Chandrababu Naidu, the then Chief Minister of Andhra Pradesh in the Preamble to the Andhra Pradesh State Population Policy. He translated this political commitment into action by setting sterilization targets and implementing a rigorous and continuous monitoring system to review achievements. The highest number of family-planning operations (800,000) performed each year was achieved during his tenure and continued to stay at about 800,000 sterilizations per year for a decade.

Female sterilization is the most used method of birth control in Andhra Pradesh. Sterilization comprised 38.3% in 1992-1993, 52.7% in 1998-1999, and 62.9% in 2005-2006 of all contraceptive methods reflecting the strong measures adopted by the Government for promoting sterilization (Table 7).

So successful was the government population-control drive that it influenced reproductive intentions of the women and resulted in the enhanced need for small families. In 1992-1993, 64.8% of women who had two children stated that they did not want any more children. This figure rose sharply to 83.7% at the time of NFHS 2 (1998-1999) and to 91.5% at the time of NFHS 3 (2005-2006). This shows the steadily-growing change in attitudes of women over a 14-year period. Even women who had only two daughters and no sons did not want any more children, indicating that son preference and sex composition of children are a lower priority for women in Andhra Pradesh compared to other states. The Government of Andhra Pradesh uses the above data to claim that its strictly-implemented family-planning programme was responsible for the low TFR and the decline in growth rate.

There is a high level of awareness about female sterilization in the state. Results of a survey on expanded and informed contraceptive choice in two districts of Andhra Pradesh in 2001 showed that most women were aware of female sterilization (19). In a recent study on demographic transition in Andhra Pradesh, the current contraceptive-use rate was 67.4%, and most of it was female sterilization (20).

However, the huge success in stabilization of the population was not replicated in the area of reduction in maternal mortality. There are no policy documents or rigorous implementation in the case of maternal health. Although the Andhra Pradesh Population Policy intended to reduce the MMR, this intention was not translated into action since very little systematic and sustained action was taken to reduce maternal mortality subsequent to the formulation of the policy. The RCH I programme tried many interventions but no concerted efforts were made to provide comprehensive maternal care services. Of late, there is stress on institutional deliveries, indicating the narrow perspective that reduces comprehensive maternal health services to childbirth in hospitals.

The level of government monitoring of a programme is an indicator of political commitment in that area. Reporting and analysis of maternal deaths are not given high priority either in performance reviews of health workers or in health statistics. Strict monitoring and reporting was implemented throughout the state for family-planning services. Bhatia showed that only one-third of identified maternal deaths were entered into the PHC and Subcentre records (10). The study by the IIHFW in 1997 reported that only 9 of 132 confirmed maternal deaths were found in official records (13).

In an effort to audit maternal death, the Government prepared a plan for each medical college to undertake verbal autopsies of maternal death reports that they received from the district. Financial provision was made for their travel and expenses through NRHM funds. This plan has not been implemented. Ad-hoc action is, however, taken on individual officers or healthcare providers when there is a media report of a maternal death in a hospital, usually a government hospital. No long-term measures are initiated and followed up.

#### Inadequate efforts at strengthening peripheral facilities for maternal healthcare

For the first four decades after 1947, the Government focused on building infrastructure, district and block hospitals, teaching hospitals, PHCs, and Subcentres. Table 3 indicates this growth in facilities. First, the priority to family planning de-emphasized maternal health. Second, although the decision to have round-the-clock service was taken more than 10 years ago, adequate personnel were not posted to opertionalize the centres. Third, training of TBAs was given high priority in the early 1990s, and outreach care was emphasized. Fourth, the focus on strengthening the FRUs undermined the potential of PHCs and relegated them to referring centres.

As the family-planning programme took precedence, the PHCs were strengthened more for operations of tubectomy than for routine maternal and child health and treatment. The PHCs that had facilities to conduct tubectomy operations were called ‘service centres'. Women came to the PHCs for sterilization after having their childbirth in the home, usually with the help of TBAs or relatives. On the designated ‘operation day', a team of highly-qualified and skilled personnel came to the PHC to deal with the additional workload. The lone staff nurse in the PHC became linked, in the minds of the public, with family-planning surgery. The decision to convert 450 of the 1,300 PHCs in 1997 into 24-hour service centres indicates that most PHCs had become non-functional, by then, due to inadequate support and monitoring. Most of these 450 PHCs did not conduct even one delivery, on an average, per day. Results of community studies indicate the unwillingness of people to use these facilities for maternal emergencies.

With the creation of the Andhra Pradesh Vaidya Vidhana Parishad in 1986 for secondary-level hospitals, the focus shifted to hospitals. Nearly 200 community hospitals, area hospitals, and district hospitals were strengthened with new buildings, facilities, and equipment. Members of the staff were trained through a rapid in-service training programme. Although the scheme was conceived with lofty intentions, in reality, it widened the distance between the primary health facilities and the referral hospitals because the two came under different administrative structures from state-level downwards.

Promotion of institutional deliveries became a major area of focus for Andhra Pradesh during the last decade due to successes observed in Tamil Nadu and Kerala. A provision was made to give additional honourarium to the staff to encourage round-the-clock childbirth services at the PHCs and CHCs. As part of this scheme, at least one medical officer, a nurse, and a cleaner were to be available beyond normal working hours. This also did not materialize due to the shortage of staff and poor monitoring. Facilities were not strengthened in the labour-rooms, and overall improvement of the PHCs did not take place.

With a focused programme strictly implemented and monitored, the story of the family-planning programme by the state within a short time could perhaps be replicated for achieving childbirths in institutions rapidly. However, the quality of care is a major concern. The PHCs in Andhra Pradesh today are not ready to handle the increased demand for services generated by higher awareness and monetary incentives. The increasing number of childbirths in private institutions is an outcome of this problem. The case study of Medak is a clear proof of the poor functioning of PHCs designated as round-the-clock service centres. The state appears to be fumbling to design and implement a comprehensive maternal and child health strategy.

#### Low focus on midwifery

Midwifery is critical to maternal health in any society because 85% of pregnancies are normal and usually end in safe outcome for the mother and the baby. The presence of a cadre of skilled midwives is conducive to the provision of quality maternal health services during a normal childbirth and timely referral for women with complications leading to reduction of maternal deaths.

Midwives were available in India at the time of independence and were active in maternal and child health. A cadre of midwifery tutors was also present. Later, midwifery teaching became integrated into the general nursing syllabus and gradually became invisible in designation, teaching or practice profession. Those working in labour-rooms became known as labour-room nurses, maternity nurses, obstetric nurses, or simply staff nurses. In the periphery, the ANM was the key service provider for maternal and child health services in the periphe-ry—PHC and Subcentres. The midwifery component of her role gradually became diluted. The process of de-skilling nurse-midwives in the periphery is associated with the stress on family planning. This decline in midwifery and the poor quality of maternal health are closely linked.

Two policy changes in the country during the 1970s resulted in the drastic decline in the quality of midwifery training and practice. The need for speeding up implementation of the family-planning programme meant that many more health centres and ANMs were required rapidly. To suit these needs, the Indian Nursing Council revised the ANM syllabus and reduced the duration from 24 months to 18 months. Private organizations were allowed to start ANM training schools. There was a mushrooming of ANM training schools in Andhra Pradesh from 14 in the 1970s to 114 in the late 1980s. In the early 1990s, one-third of the schools for training multipurpose health workers in the country were located in Andhra Pradesh. Most of these had no faculty, classrooms, or demonstration laboratories to teach maternal and child health.

The new cadre was called Multipurpose Health Worker (MPHW) (F). The existing ANMs were given a short orientation training and were redesignated as MPHWs (F). The nursing and midwifery component of the ANM became invisible not only in their new designation but in the loss of skills and functions. They were told that achievement of the family-planning targets, with focus on female sterilization, was their main responsibility, especially since they were paid from family-planning funds. Strong action was taken if they did not achieve the monthly target of family-planning cases but no action was taken if they did not assist in deliveries or visit postnatal mothers. As a direct outcome of this policy shift, community perception of ANMs changed from maternal and child healthcare providers to family-planning workers.

Technical supervision at the field level also became diluted. Public-health nurses (PHNs) as a cadre with higher qualifications were discontinued due to union pressure in 1990, and health visitors were promoted as PHNs without further training and designated as PHN (non-teaching). The course for the Lady Health Visitors for two and a half years was discontinued in the 1970s, and a six-month promotional course was started according to the Indian Nursing Council (INC) guidelines. This course was further shortened to six weeks in Andhra Pradesh leading to deterioration of nursing and midwifery supervision in rural areas.

Results of a study of nursing and midwifery in Andhra Pradesh in 2005 showed that nursing schools were inadequately equipped with models, books, and teaching-aids (21). Teachers had lost most basic skills of midwifery and pedagogy. As a result of inadequate support and gradual dilution, midwifery training and practice today are of low quality. There is no evidence of commitment of the state for improving the quality of midwifery services. Training of staff nurses is underway in the state as part of the SBA training of the Government of India but the quality and skills are inadequate. In 2006, the Government of Andhra Pradesh, in collaboration with Academy for Nursing Studies, opened a Centre for Advanced Midwifery Training. Tutors received in-service training, and some focus was laid on midwifery strengthening. However, there is no move in the state to start a midwifery cadre or to more efficiently use the staff nurses for childbirths and maternity services in the PHCs.

#### Low status of women has not generated demand for quality maternal health service

Around 18% of girls aged 15-19 years were already mothers or pregnant in Andhra Pradesh at the time of interview according to the NFHS 3. The median age at first birth for women aged 25-49 years, who were interviewed as part of the three NFHS surveys, showed only marginal improvement. It was 17.9 years in 1992-1993, 18.0 years in 1998-1999, and 18.8 years in 2005-2006, indicating that progress in the reproductive pattern of women has changed only in terms of the number of childbirths and not in other areas (Table 8).

**Table 8.** Trends in age at marriage in Andhra Pradesh, 1992-2006

Indicator	NFHS 1 1992-1993	NFHS 2 1998-1999	NFHS 3 2005-2006
Percentage of women aged 20-24 years married by 18 years of age	68.6	64.3	54.7
Median age (years) at first birth for women aged 25-49 years	17.9	18.0	18.8

NFHS=National Family Health Survey

Data from the recent study on demographic transition in Andhra Pradesh cited earlier showed that there is a slight shift in age at marriage in the state (20). This gives an impression of early stages of nuptiality transition in Andhra Pradesh.

Andhra Pradesh lags behind most Indian states in terms of female literacy. While 53.67% of females aged seven years and above are literate in the country, only 50.43% are literate in the state (Table 9). Andhra Pradesh lags far behind the other three southern states (Karnataka, Kerala, and Tamil Nadu) in the percentage of females aged above seven years who are literate according to the Rural Health Statistics Bulletin of 2007.

**Table 9.** Female literacy rates (%) in Andhra Pradesh and southern states

Year	Andhra Pradesh	Karnataka	Tamil Nadu	Kerala	All-India
1981	24.16	33.16	40.43	73.36	29.84
1991	33.71	44.34	52.29	86.17	39.37
2001	50.43	56.87	64.43	87.86	53.67

The 1990s witnessed a high level of women's activism with the launching of a sustained antiliquor movement (1994). Participation in the International Conference on Population and Development (1994) and in the Fourth World Conference on Women (1995) also created higher awareness among women. The main political opposition—Telugu Desam—led by N.T. Rama Rao and Chandrababu Naidu—supported the women's movement and made promises to prohibit liquor and take up overall development of women, along with higher political participation. When they succeeded in the election, they fulfilled some of these promises. The Andhra Pradesh State Women's Policy (1996) was, therefore, formulated as a result of activism and political commitment. A three-day state-level consultation was held with participation of several national experts. The policy also included a women's health component that placed stress on enabling women to seek health services. The decision to convert one-third of the PHCs to Women Health Centres to provide round-the-clock services was an outcome of this consultation. However, no major focus was laid on overall improvement of the health of women in the state, and the women's health policy was not actually finalized. The State Commission on Women gradually reduced its functions.

Anaemia is a major problem among women in Andhra Pradesh. Compared to only 22.6% ever-married men aged 15-49 years who were anaemic, a higher percentage (62.0%) of women of the same age and marital status was anaemic according to the NFHS 3, indicating the widespread gender disparities in food consumption. The *Anganwadi* centres operated by the Women's Development and Child Welfare department could not cope with improving the nutrition of women despite several years of implementation. Efforts at increasing women's use of public-health facilities for maternal health also did not show much success. The *Mahila Swasthya Sanghs* and mothers' committees largely remained paper activities. Linkages were not built with self-help groups, resulting in lost opportunity to influence the health of women.

### Conclusions and future action

Andhra Pradesh achieved great success in development and demographic fields in the last two decades. It had the lowest decadal growth rate, the highest number of tubectomies performed on women, the largest number of women's self-help groups, the first state population policy, etc. At the same time, the state has not been successful in overall development of women or in providing healthcare to women and children, indicating gross paradoxes in development of the state.

The single focus on stabilization of the population resulted in negligence in broad primary healthcare. Andhra Pradesh presents perplexities and paradoxes relating to social development, gender, and use of health services. The state proved that it is highly successful at conceptualizing, systematically implementing, and rigorously monitoring the programme that it intends to achieve—slower population growth. The visible demographic transition in Andhra Pradesh is the result of this close match between political will and programme implementation. Maternal health has not yet received the focus of the Government, except on an ad-hoc basis and on a very narrow level of promoting institutional deliveries. The relatively-slow decline in the MMR in Andhra Pradesh compared to the rapid decline in population growth is the result of skewed political priority setting and selective programme implementation.

The state, therefore, provides fertile ground for advocacy towards concrete and sustained policies and programmes for safe motherhood. The success of the family-planning programme was due to political will and bureaucratic commitment at the highest level. Advocacy effort, therefore, needs to be concentrated at the highest political level in the state. Examples from the family-planning programme need to be reviewed, analyzed, and used for implementing a safe motherhood programme. Several NGOs and networks brought up the issue of high maternal mortality during government meetings.

As a result of this case study, the Academy for Nursing Studies is collaborating with the Government of Andhra Pradesh to develop a model in Medak for operationalizing the PHCs as round-the-clock service centres for maternal and neonatal care by enhancing the midwifery of nurse-midwives in the periphery. The PHC doctors, staff nurses, and ANMs are actively involved in this project. The tracking of maternal deaths taken up through this case study in 2006-2007 has led to further advocacy at the state level and has developed links with other advocacy interventions. As part of this, 30 cases of maternal death were selected from all over the state and were analyzed from a rights and legal framework for advocating with the State Government to strengthen its strategies for reduction in maternal mortality.

## ACKNOWLEDGEMENTS

The author acknowledges the deep gratitude to Mr. Khaja Saleemuddin and Dr. Francis Raj for data collection and compilation and to Ms Sujatha and Mrs Olivia Benjamin for collecting data on maternal deaths and for coordinating assessment and interventions in Medak. The cooperation and support of the different departments of the Government of Andhra Pradesh is also appreciated.
